# Efficacy and safety of oral immunotherapy for peanut, cow's milk, and hen's egg allergy: A systematic review of randomized controlled trials

**DOI:** 10.1002/clt2.12268

**Published:** 2023-07-01

**Authors:** Caroline J. Lodge, Nilakshi Waidyatillake, Rachel L. Peters, Merryn Netting, Xin Dai, John Burgess, Catherine J. Hornung, Kirsten P. Perrett, Mimi L. K. Tang, Jennifer J. Koplin, Shyamali C. Dharmage

**Affiliations:** ^1^ Allergy and Lung Health Unit Melbourne School of Population and Global Health The University of Melbourne Carlton Victoria Australia; ^2^ Centre for Food and Allergy Research Murdoch Children's Research Institute Parkville Victoria Australia; ^3^ Department of Pediatrics The University of Melbourne Parkville Victoria Australia; ^4^ Women and Kids Theme South Australian Health and Medical Research Institute (SAHMRI) Adelaide South Australia Australia; ^5^ Discipline of Paediatrics University of Adelaide Adelaide South Australia Australia; ^6^ Department of Allergy and Immunology Royal Children's Hospital Melbourne Victoria Australia; ^7^ Allergy Immunology Murdoch Childrens Research Institute Parkville Victoria Australia; ^8^ Child Research Centre University of Queensland South Brisbane Queensland Australia

**Keywords:** desensitization, food allergy, oral immunotherapy, randomized controlled trials, sustained unresponsiveness

## Abstract

**Background:**

Oral immunotherapy (OIT) is a promising treatment for food allergies; however, safety is a concern. We synthesized evidence from the best randomized controlled trials (RCTs) on efficacy/safety of OIT for desensitization (DS) and remission (sustained unresponsiveness (SU)) in IgE mediated allergy to peanut, hen's eggs, and cow's milk.

**Body:**

We searched Pubmed, EMBASE, and Cochrane databases (Until Oct 22) identifying 16 eligible RCTs published in English measuring food allergy by food challenge at the beginning and at the end of the study. The Cochrane Risk of Bias tool was used to assess study quality. We found 18 eligible studies. There was evidence of efficacy for DS for all allergens: peanut (RR 11.32; 95% CI 5.93, 21.60, *I* ^2^ 49%, 8 studies); hen's egg (RR 4.67; 2.66, 8.21, *I* ^2^ 0%, 5 studies); cow's milk (RR 13.98; 3.51, 55.65, *I* ^2^ 0%, 4 studies) and evidence for SU for peanut (RR 7.74; 2.90, 20.69, *I* ^2^ 0%, 3 studies) and hen's egg (RR 6.91; 1.67, 28.57, *I* ^2^ 0%, 2 studies). Allergic events were increased with intervention, and risk of adrenaline use increased for peanut RR 2.96; 1.63, 5.35, *I* ^2^ 0%, 8 studies; egg RR 1.71; 0.42, 6.92, *I* ^2^ 0%, 6 studies; and milk RR 8.45; 2.02, 35.27, *I* ^2^ 0%, 4 studies.

**Conclusion:**

We found strong evidence that peanut, hen's egg, and cow's milk OIT can induce DS and some evidence for remission. There was a high risk of allergic reactions. Generalizability to the entire food allergic population is not known.

## 1 INTRODUCTION

IgE‐mediated food allergy affects up to ten percent of 12‐month old children^1^ and has a significant impact on quality of life (QoL) of the child and their family.^2^ Egg, milk, and peanut allergies are among the most common food allergies.^3^ The causes of food allergy remain poorly understood; however, there is evidence that early introduction of allergenic foods may help prevent the development of food allergy.^4^, ^5^ Current management of established food allergies includes strict allergen avoidance and early management of reactions to accidental ingestion. In practice, total avoidance is difficult and most children experience multiple episodes of ingestion.^6^ Food avoidance impacts the quality of life of the child and their family.^7^ Finding a ‘cure’ for food allergy remains a high priority.

Food allergen immunotherapy is receiving increased interest as a potential treatment. To date, the most promising method is oral immunotherapy (OIT).^8^, ^9^ OIT regimens vary but usually involve three phases: initiation, escalation, and maintenance. In practice, this means ingestion of food allergens starting from a very small amount (initiation) and incrementally increasing over a defined period (escalation) until a target dose is achieved and maintained (maintenance). Outcomes of OIT are usually measured as desensitization (DS) and sustained unresponsiveness (SU) (remission).^10^ DS describes increase in reaction threshold, that is, the amount of allergen able to be orally ingested without reaction. It is a temporary state maintained with continued allergen exposure^11^ typically daily. Remission describes lack of clinical reactivity which is maintained for a time despite discontinuing allergen ingestion and is indicative of a longer lasting change. There is no consensus on the time required without allergen intake to define remission but periods between 2 weeks and 6 months have been applied in published studies.^10^

The main concerns regarding OIT are the potential for frequent/daily OIT doses to cause life‐threatening allergic reactions^12^, ^13^, ^14^ and uncertainty about impact on quality of life (QoL). There is also continued uncertainty about the potential for and rate of long‐term remission from these therapies. Improved understanding of OIT risks and benefits^15^ and whether it leads to improved QoL remains to be confirmed.^16^, ^17^

Several systematic reviews have addressed OIT as a treatment for peanut, egg, and/or milk allergy.^12^, ^18^, ^19^, ^20^, ^21^, ^22^ Nurmatov et al's^18^ systematic review included 25 RCTs and 6 non‐randomized studies published up to March 2016. A limitation of this systematic review was the pooling of heterogeneous studies and methods. Further issues were inclusion of studies that did not determine food allergy in all participants using OFC at entry, and failure to distinguish between intention to treat (ITT) and per protocol (PP) analyses, with inclusion of both in meta‐analyses. The Cochrane review published in 2018^19^ focussed on egg OIT. It comprised 10 RCTs of OIT for eggs and did not report outcomes of desensitization to a fixed dose or sustained unresponsiveness, instead reporting any tolerance to a part amount or a “serving size”. A more recent OIT systematic review of RCTs published in the Lancet focused only on peanut.^12^ It comprised 12 trials but included 5 which had not measured peanut allergy by OFC at baseline and 1 using sublingual immunotherapy as the control. In 2022, 3 new systematic reviews were published^20^, ^21^, ^22^ with varying findings for the efficacy of OIT.

Although informative, none of these reviews have been limited to the best evidence, namely good quality RCTs where food allergy was established objectively by oral food challenge (OFC) at the beginning and end of the trial for both the intervention and control groups, and intention‐to‐treat (ITT) analyses reported. It is critical to limit to best quality RCTs to obtain accurate information concerning efficacy and safety to provide the most unbiased estimates of the risks and benefits of using these treatments. We aimed to review the current best evidence from RCTs for the effectiveness of peanut, egg, and cow's milk OIT on desensitization and remission and adverse allergic events.

## 2 METHODS

### 2.1 Search strategy

PubMed, EMBASE and Cochrane databases searched from inception for peer‐reviewed English publications (Tables S1–S3). PROSPERO systematic review registry (No: CRD 42018099929). Last search Oct 2022.

### 2.2 Inclusion criteria

RCTs, published in English, investigating OIT efficacy for OFC‐proven IgE mediated food allergy to peanut, egg, or milk. Intervention: OIT. Comparison: placebo or food avoidance.

Included studies were required to have outcomes of desensitization (DS) and/or sustained unresponsiveness (SU) (terminology used in included studies for remission) in humans, to have OFC on all participants prior to commencement and on study completion. ITT reported or able to be calculated.

### 2.3 Outcomes of interest

1. Desensitization (DS) is defined as increase in reaction threshold for allergens.
2. Sustained Unresponsiveness (SU) is defined as a lack of clinical reactivity maintained for at least 2 weeks despite allergen discontinuation.
3. Adverse reactions are any reported adverse reactions in either placebo or treatment groups.

### 2.4 Selection of studies

After the removal of duplicates, studies identified were independently screened by two authors (NW, CL). Full‐texts were then read by the same authors to determine suitability for inclusion. Any disagreements were settled by a third author (SD). Endnote and Covidence were used.

### 2.5 Data extraction

Using a standardized form, data were extracted independently by 2 authors (NW, MN, JB or CL). We extracted trial details: (first author; year published; country; number and age of participants; duration; how food allergy defined, consideration of baseline reaction threshold; outcomes and their definitions; confounding and moderating factors, numbers (proportions) achieving DS/SU in the treatment and control arms (ITT)); details of immunotherapy: (starting dose; dose escalation; final dose; length of treatment; need for hospitalization/observation as part of regime and any immunomodulatory therapies added) and adverse effects (any vs. no reactions per child (trials with placebo); and use of parenteral adrenaline for intervention versus control (placebo or avoidance)).

### 2.6 Quality assessment/risk of bias

Assessed independently by 2 authors (XD or CL) using the Cochrane risk of bias tool for RCTs.

### 2.7 Statistical analysis

Associations from individual studies expressed as proportions achieving DS and SU were converted to risk ratios and 95% CIs and pooled in random effects (inverse‐variance model) meta‐analyses for individual allergens (peanut, egg, and milk). *I* ^2^ statistic was used to assess heterogeneity with values >75% considered high.^23^ Effect estimates for adverse reactions pooled. All analyses used Stata/SE 17.0.

We calculated the number needed to treat (NNT) for DS, SU, and allergic adverse events as per Cochrane methods. NNT = 1/[(RR‐1) x risk in control group] for RR > 1 and, NNT = [1/(1‐RR) x risk in the control group] for RR < 1.^23^ The assumed control risk (ACR) was calculated from the numbers in the included studies.

### 2.8 Role of funding source

The funder had no role in the study design, collection, analysis, or interpretation of the data nor in the decision to submit for publication.

## 3 RESULTS

**FIGURE 1:**
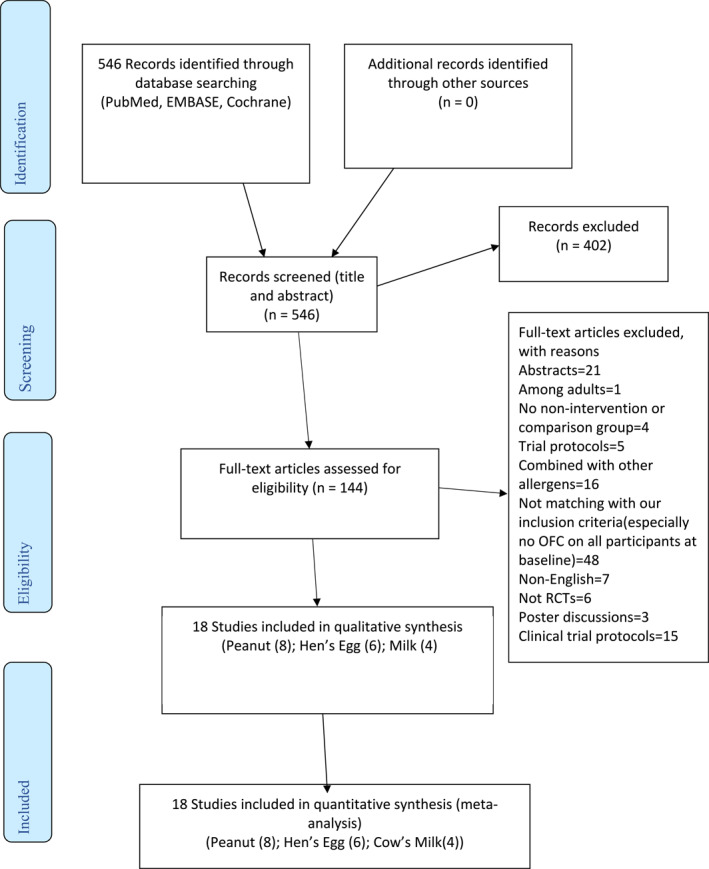
PRISMA flow diagram of study selection.

Of the retrieved 546 papers (Figure 1), 144 had full text screening and 18 were included (Figure 1). These 18 RCTs comprised eight peanut, six hen's eggs, and four cow's milk OITs. All RCTs measured DS, with five also measuring SU.^11^, ^24^, ^25^, ^26^, ^27^

### 3.1 Peanut OIT

#### 3.1.1 Overview of studies

The eight peanut RCTs^11^, ^26^, ^27^, ^28^, ^29^, ^30^, ^31^, ^32^ included 1414 participants in total with numbers varying between 56^29^ and 555^32^ participants. One study was conducted in the UK,^28^ one in Germany,^30^ three in the USA,^11^, ^27^, ^29^ two in multi‐country sites^31^, ^32^ (Europe and US) and one in Australia (Table 1).^26^ Participant age ranged from a mean of 3.1^27^–12.4 years.^28^ Age inclusion criteria varied with four studies^28^, ^30^, ^31^ including participants up to 16 or 17 years and two^11^, ^29^, ^32^ up to 21 years. Four studies^26^, ^27^, ^29^, ^31^ excluded children with previous life threatening or severe anaphylaxis and seven studies^11^, ^26^, ^27^, ^28^, ^29^, ^30^, ^31^ excluded children with severe or poorly controlled asthma. Importantly, five of the studies^11^, ^26^, ^29^, ^30^, ^32^ provided detailed information on the reasons screened participants were not included in the studies. Of the 1495 people screened in these 5 studies, 205 (14%) were excluded because they were tolerant of peanut on the initial DBPCFC (Table 2).

**TABLE 1:** Overview of included studies.

First author, year, country	Population/& recruitment location	Inclusion & exclusion criteria	Sample size trial arms & age	Randomisation	Intervention	Outcome definition	Outcomes	Adverse events & adrenaline use	Author conclusion comments
Peanut									
**Anagnostou** 2014 Cambridge UK STOP II DS	NIHR/Wellcome Trust Cambridge clinical research Facility 2‐Phase RCT‐ recruited locally and nationally	*Inclusion:* 1. 7–16 years 2. Immediate hypersensitivity after peanut ingestion 3. Pos SPT peanut ≥3 mm 4. Pos DBPDFC *Exclusion:* 1. Major chronic illness (asthma, eczema, excepted) 2. Household member allergic to peanuts 3.Unwillingness or inability to comply	*Total:* 99 *OIT:* 49 *Control:* 50 Age: 7–16 yrs (median 12.4)	*Method:* Audited on‐line system Randomizer, Medical University of Graz, Austria) minimization used based on baseline characteristics with weighting prob 0.8‐ (sex, age, challenge threshold, sIgE, severity, asthma, other food allergy) *Blinding:* None	*Intervention:* Peanut flour(light roast flour) Golden Peanut Company, Alphretta, GA, USA *Control:* Avoidance *Regimen:* mixed *Duration:* 6 months *Maximum OIT dose:* 0.8 gm peanut protein daily (5 peanuts)	**Desensitization** *Definition:* Neg DBPCFC *Cumulative dose*: 1.4 gm peanut protein (approx. 10 peanuts) *Tested:* 6 months	**Desensitization** *ITT* OIT: 24/49 (49%) Control: 0/50 (0%) PPA (with censoring and loss to FUP) OIT 24/39 Control 0/46	**Any** Only reported for intervention and not separately for 2 study phases Common, primarily GI Severe OIT 21/49 (22%) wheeze/laryngeal oedema (0.41% of doses) **Adrenaline** OIT 1/49 C Not available **EoE** Not reported	OIT successful for most children for desensitization to clinically meaningful threshold
**Bird** 2018 United States DS	Peanut allergic children Enrolled from 8 US centres	*Inclusion:* 1. Age 4–26 years 2. History of peanut allergy 3. IgE ≥0.35KUA or SPT ≥3 mm in past 12 months 4. DBPCFC+ at or before 100 mg single dose peanut protein (143 mg cum) (PRACTALL guidelines) *Exclusion:* 1. Hx of CVD 2. Frequent or life threatening anaphylaxis, 3. Eosinophilic gastrointestinal disease, 3. On other intervention 4. Other chronic illness—(except asthma, eczema, rhinitis) 5. Severe or uncontrolled asthma 6. Use of specific medications	*Total:* 56 *OIT:* 29 *Control:* 27 Age: *OIT* 4–21yrs median 7 *Control* 4–14yrs (med 8)	*Method:* 1:1 using central randomization schedule of randomly permuted blocks‐ independent statistician *Blinding:* DB	*Intervention:* ARA101‐ defatted lightly roasted peanut flour (capsules) *Control:* Placebo (oat flour) *Regimen:* mixed *Duration:* 5–10 months (average 5.5) *Maximum OIT dose:* 300 mg peanut protein	**Desensitization** *Definition:* Neg DBPCFC single dose of 300 mg (443 mg cumulative) of peanut protein PRACTALL guidelines	**Desensitization** *ITT* OIT: 23/29 (79%) Control: 5/27 (19%)	**Any** OIT 28/29 (26 treated) C 22/26 (10 treated) Primarily GI **Adrenaline** OIT 1/29 C 0/27 EoE 1/29 in OIT gp/resolved off treatment	
**The Palisade group** 2018 PALISADE Nth Amercia & Europe DS	66 sites in 10 countries in North America and Europe	1. 4–55 years 2. Clinical Hx peanut allergy 3. SIgE ≥0.35 kUA 4. SPT 3 mm > control 5. DBPCFC to up to 100 mg peanut protein (PRACTALL)	*Total:* 555 Then excluded >17 (55 people) So‐ 496 (3–17years) *OIT: 372* *Placebo:* 124 *Age* 4–17 yrs	*Method:* random assigned 3:1—central randomization in randomly permuted blocks, interactive online system *Blinding:*DB	*Intervention:* AR101 *Contol:* Placebo *Regimen:* Gradual *Maximum OIT dose:* 300 mg daily *24 week maintenance*	Single dose at least 600 mg	**Desensitization** OIT: 250/372 (67%) Placebo 5/124 (4%)	**AEs** **Any** OIT 367/372 98.7% C 118/124 95.2% Severe OIT 8 4.3% C 1 0.8% (9 severe events in 8 participants vs. 1) **Adrenaline** OIT: 52/372 C 8/124 Overall serious or adverse events **EoE** OIT 1/372	No significant effect in patients 18 ‐ 55
**Chinthrajah** 2019 POISED US DS SU	DBPC Parker Centre for Allergy and Asthma research—Stanford University Adult and pediatric patients aged 7–55 yeas	*Inclusion:* 1.DBPCFC (≤500 mg peanut protein 2.+ve SPT ≥5 mm 3.sIgE >4kU/L Age 7–55yrs *Exclusion:* 1.Severe/uncontrolled asthma 2.Eosinophilic gastrointestinal disease 3.Sensitivity to oats	*Total:* 120 age 11yrs (iqr 8–15) *OIT:* i)60 (OIT then 0 gm) ii)35 (OIT then 300 mg/day) *Control:* 25 Age: OIT 4–21yrs med 11 yrs Control 4–14yrs (med 8)	*Method:* 2 × 2 block design into 3 arms 2.4:1.4:1 *Blinding:* DB	*Intervention:* ARA101‐ defatted lightly roasted peanut flour (capsules) *Control:* Placebo (oat flour) *Regimen:* ? *Duration:* 24 months then 12 months off OIT *Maximum OIT dose:* 4000 mg peanut protein	**Desensitization** *Definition:* Neg DBPCFC *Cumulative dose*: 4000 gm(16–18 peanuts) *Tested:* 104 weeks **Sustained Unresponsiveness** *Definition:* Neg DBPCFC *Cumulative dose*: 400 gm(16–18 peanuts) *Tested:* 117, 130, 143, 156 weeks	**Desensitization** ITT Peanut‐0 versus Placebo 51/60 (85%) versus 1/25(4%) **Sustained unresponsiveness** ITT Peanut 0 versus placebo 117 21/60(35%) versus 1/25(4%) 130 12/60 (20%) versus 1/25(4%) 143 9/60 (15%) versus 1/25 (4%) 156 8/60(13%) versus 1/25 (4%)	Statistics for first 12 months **Any** OIT Peanut 0–57/60%‐95% 53/60 had grade 1 reactions(88%) Resp 34 (57%) Peanut300 32/35 (91%) 30/35 grade 1 (86%) Resp 18(51%) Placebo 16/25(64%) 13/25 (52%) grade. Resp 6 (24%) **Adrenaline** OIT 18/95 C 0/25 **EoE** ‐ 1/120 (OIT) 2% SAE rate in intervention arms	No differences, adults versus children Higher baseline sIgE, Arah1&2 IgE associated with failure Lower basophils at baseline associated with success Increased sIgG4/sIgE assoc with success
**Blumchen** 2018 Germany DS	7 German sites‐ outpatient clinics and tertiary care clinics (consecutive recruitment)	*Inclusion:* *1.Age3‐17yrs* 2. sIgE>0.35kU/L 3. Challenge proven peanut allergy—Open OFC 4. Parents could understand/follow emergency instructions *Exclusion:* Participation in another trial Any other form immunotherapy Severe disease (eg uncontrolled asthma)	*Total:* 62 *OIT:* 31 *Control:* 31 *Age:* *OIT:* 6.6 (4.8–9.8 IQR) *Control:* 7.9 (4.6—10.7IQR)	*Method*: 1:1 block randomization size 4 (Dat Inf, rand List, version 1.2) Stratified by age (>or<6 yrs) and sIgE (, or > 50 kU/L). Independent statistician *Blinding:* DB	*Intervention:* Light roasted peanut flour (Byrd Mill Company‐ Ashland, Va) *Control:* Placebo Regimen:mixed Duration: 14 months *Maximum OIT dose:* 125–250 mg peanut protein	**Desensitization** *Definition:* *Open OFC* Neg DBPCFC single dose of 300 mg (443 mg cumulative) of peanut protein PRACTALL Guidelines *Tested:* 16 months	**Desensitization** ITT OIT: 23/31 (74.2%) Control: 5/31 (16.1%)	**Any** OIT 27/311‐10 (90%) Control 24/31 (77%) **Adrenaline** No Adrenaline used **EoE** none found AEs more common with intervention doses 83% of intervention versus 45% of placebo	Low‐dose OIT is a promising, effective and safe option for peanut allergic children, leading to improvement in QoL, a low BOT, and immunologic changes showing tolerance development
**Hourihane** 2020 ARTEMIS Europe DS	18 hospitals in Ireland, France, Germany, Italy, Sweden and UK	*Inclusion:* 1.Age 4–17 yrs 2. Clinical Hx peanut allergy 3. SPT≥3 mm and/or sIgE. ≥0.35kU/L 4.+ve DBPCFC 5.Sx at ≤ 300 mg peanut protein (1 peanut) *Exclusion* 1.Severe/life‐threatening anaphylaxis within 860 days 2.Severe/uncontrolled asthma 3.Hx of eosinophilic oesophagitis or chronic GI Sx	*Total:* 175 *OIT:* 132 *Control:* 43	*Method* Randomly assigned 3:1 in blocks of 8‐ proprietary interactive web response system using computerized random number generator *Blinding:* DB	*Intervention* AR101 *Control:* Placebo *Regimen:* Mixed *Duration:* 9 months *Maximum OIT dose:* 300 mg peanut protein	**Desensitization** *Definition:* Neg DBPCFC *Cumulative dose:* 1000 mg PRACTALL *Tested* 9 months	**Desensitization** ITT OIT: 77/132 (58%) Control: 1/43 (2%)	Any OIT 130/132 C 42/43 OIT: Mild 66 (50%) Mod 63 (48%) Sev 1(1%) Control: Mild 24 (56%) Mod 18 (42%) Sev 0 (0%) Adrenaline OIT 9/132(7%) C 1/43 (2%) No EoE during trial	AR101 led to rapid desensitization with predictable safety profile and improved food allergy related QoL for caregiver and participant
**Jones** 2022 IMPACT USA DS SU	5 Academic medical centres in USA. (Arkansas, Johns Hopkins, Mount Sinai, Stanford, University of Nth Carolina).	*Inclusion:* 1. Age—12 to 48 months 1.Clinical Hx peanut allergy 2. sIgE ≥5KU_A_/L, 3.SPT ≥3 mm neg control 4.+ve DBPCFC to cumulative dose ≤500 mg *Exclusion:* 1. severe peanut anaphylaxis 2.Asthma more than mild 3. Uncontrolled asthma 4.Uncontrolled AD 5.Eosinophilic GI disease	*Total: 146* *OIT: 96* *Control: 50* *Age:* 12–48 months of age. *Median age ‐* 39.3 months (IQR 30.8–44.7 months) 68% Male	*Method:* Randomly assigned (2:1) *Blinding:* DB	*OIT*‐Lightly roasted, partly defatted peanut flour *Control:* placebo.(oat flour) *Regimen:* mixed *Duration*: 134 weeks. Then 26 weeks avoidance *Maximum OIT dose*: 2000 mg peanut protein/day	**Desensitization** (**primary outcome)** *Definition:* ‐ve DBPCFC *Cumulative dose:* 5000 mg peanut protein *Tested* 134 weeks (30.8 months) **SU** *Definition* *Cumulative dose:* 5000 mg peanut protein *Tested* 160 weeks(36.8 months) After 6months(26weeks avoidance)	**Desensitization** ITT OIT: 68/96 (71%, 95% CI 61–80) **Control** 1/50 (2%, 95% CI 0.05–11) Risk difference 69% (95%CI 59%–79%} Risk ratio 35 (95% CI 5.1–248) **Sustained unresponsiveness** ITT OIT: 20/96 (21%, 95% CI 13–30) Control: 1/50 (2%, 95% CI 0.05–11) Risk difference 19% (95% CI 10%–28%} Risk ratio 10.4 (95% CI 1.4–75)	**Any** OIT: 94/96 (98%) Placebo: 40/50 (80%) **Adrenaline**: Peanut OIT: 21/96 (22%) Placebo: 0/50 “Dosing and challenge reactions were expected and not reported unless hypotension, cyanosis, o2 sat <92, confusion, collapse, loc, incontinence or required >2 epinephrine doses” **EoE** —3 in OIT gp (2 resolved off treatment)	Peanut OIT started before age 4 years in children with peanut allergy is associated with increased Peanut desensitization and remission. Window of opportunity Lower baseline IgE associated with greater chance remission
**Loke** 2022 PPOIT‐003 Australia DS SU	3 Australian tertiary hospitals. Women's and Children's Hospital, Adelaide [SA] Royal Children's Hospital Melbourne [VIC] Perth Children's Hospital [WA]	*Inclusion:* 1.Age 1–10 yrs, 2. Weight >7 kg, 3. +ve DBPCFC, 4. +ve SPT (wheal>3 mm) or peanut specific IgE ≥0.35kU/L *Exclusion:* 1. Severe anaphylaxis or during DBPCFC 2. FEV1 <85% pred 3. FEV1/FVC <85% pred 4. chronic persistent asthma 5. cardiac disease 6. Beta blockers or ACE 7 GI disorders 8. Recent surgery 9. Recent probiotics 10. Major illnesses 11. Unable to follow protocol 12. Many others	Randomized: 201 PPOIT (probiotic + peanut OIT)::79 OIT:83 Placebo:39 Age av: Total—5.9 yrs PPOIT 6 OIT 5.8 Placebo 6	*Method:* Randomly assigned permuted blocks of 5(2:2:1) Stratified by site, age >5, SPT>10 mm Blinding: DB	*Probiotic:* *Lactobacillus rhamnosus* ATCC 53103 2 × 10^10^ CFU *OIT:* Peanut: 12% defatted peanut flour *Control:* Maltodextrin *Regimen:* mixed *Duration:* 78 weeks. Then 12 more months avoidance *Maximum OIT dose:* 2000 mg peanut protein/day	**Desensitization** ‐ve DBPCFC **Cumulative dose**: 4950 mg peanut protein **SU** (primary outcome) ‐ve DBPCFCs at treatment completion & 8 weeks after treatment. **Cumulative dose**: 4950 mg peanut protein	**Desensitization** *PPOIT* 61/79 (77%) *OIT:* 61/83 (73%) C: 2/39 (5%) **Risk difference** *PPOIT vs C* 72.1%(95% CI 60.5–83.6) *PPOIT vs OIT* 3.72% (−9.5–17.0) *OIT vs Plac* 68.4% (56.6–80.1) **SU** *PPOIT* 36/79 (46%) *OIT:* 42/83 (52%) *Placebo:* 2/39 (5%) **Risk difference** *PPOIT vs Placebo* 40.4% (95% CI 27.5–53.4) *PPOIT vs OIT* −5.0% (−20.4–10.3) *OIT vs Placebo* 45.5% (32.7–58.3)	**Any** PPOIT 72/79 (91%) OIT 73/83 (88%) Control 28/39 (72%) **Adrenaline** PPOIT: 2/71 (3%) OIT 4/70 (6%) C: 0/39 (0%) **EoE**‐ 2 cases in OIT group (none in PPOIT or control) Exposure adjusted incidence of AEs PPOIT 10.58, OIT 11.36, control 2.09	Both PPOIT and OIT were effective at inducing desensitization and sustained unresponsiveness.Addition of a Probiotic did not improve efficacyof OIT, but may improve safetyAdverse events,mostly mild,were common inthe activetreatment groups.
Hen's Egg									
**Akashi** 2017 Tokyo, Japan DS	Egg allergic patients from Outpatients, National Centre for child Health & Development Threshold dose defined at beginning	*Inclusion:* 1. Egg‐specific IgE ≥0.7 U_A_/mL 2. DBPCFC pos. to egg 3. Elimination of eggs from the diet 4. Caregiver agreed *Exclusion:* Anaphylaxis (hypotension or dyspnoea on egg challenge)	*Total:* 36 (25 boys; 11 girls) *OIT:* 18 *Control:* 18 Age 3–15 years: mean 5.8	*Method:* Computerized algorithm 1:1 *Blinding:* None	*Intervention:* Hens egg whole (Dried powdered) *Control:* Avoidance *Regimen:* Gradual *Duration:* 6 months *Maximum OIT dose:* 1.7 gm EWP	**Desensitization** *Definition:* Neg DBPCFC (AAAI scoring ≥1) *Cumulative dose* 1.4 gm *Tested:* 6 months	**Desensitization** *PP* OIT: 8/14 (57%) Cont: 0/16 *ITT* OIT 8/18 (44%) Cont 0/18	**Any** OIT 17/18 C not available **Adrenaline**—none given **EoE**‐ none detected	OIT effective in increasing threshold and inducing desensitization OIT group clearly more tolerant at beginning from OFC
**Caminiti** 2015 Messina, Italy DS SU	Egg allergic patients from Allergy units of the departments of paediatrics of Messina and Catania university hospitals Inhalant allergy in 9 but no other food allergies	*Inclusion:* 1. Age ≥4 2. Demonstrated IgE‐mediated Hens egg: Clinical history; 3. HE specific IgE & SPT 4. Pos DBPCFC (3.7 gm EW protein) *Exclusion* 1.Suspected soy allergy or IgE to soy 2.Sensitized to other foods	*Total:* 31 *OIT:* 17 *Control:* 14 Age: 4–11 years (median 6)	*Method* “computer‐generated randomization list” *Blinding:* Double	*Intervention:* Hen's egg white (dehydrated) *Control:* Placebo (corn flour) *Regimen:* mixed *Duration:* 10 months (4 months OIT 6 months egg containing diet) Control group avoided HE for 9 months after trial *Maximum OIT dose:* 4 gm EWP	**Desensitization** *Definition:* Neg DBPCFC (EACCI) *Cumulative dose:* 3.7 gm egg white (equiv to 1 boiled egg) *Tested:* 4 months **Sustained Unresponsiveness** *Definition:* Neg DBPCFC Time between OIT & OFC: 3 months	**Desensitization** *ITT* 4 months OIT 16/17 Cont 0/14 **Sustained Unresponsiveness** 13 months *ITT* OIT 5/17 Cont 1/14	**Any** *OIT*: 5/17 C: 0/14 **Adrenaline** **OIT 1/17** **C 0/14** **EoE**‐ none detected	OIT effective for desensitization
**Dello Iacono** 2013 Italy DS	Severe egg allergic patients from Paediatric and Allergology Unit of the Fatebenefratelli Hospital in Benevento, Italy Severe egg allergy Reaction eliciting dose used to restrict 0.9 mL raw HE	*Inclusion:* 1. ≥1 anaphylactic reaction to accidental trace exposure to HE within 12 months pre‐enrolment 2. Previous HE specific IgE & SPT ≥ 3 mm with raw egg white 3. DBPCFC pos at ≤ 0.9 mL raw HE emulsion *Exclusion* 1.Poorly controlled asthma 2. Parents unreliable 3. Sensitized to other foods	*Total:* 20 *OIT:* 10 *Control:* 10 Age: *OIT*: 5–10 (med 6.6) *Cont*:4–11 (med 8.6)	*Method:* Computerized randomisation *Blinding:* None	*Intervention:* Hens egg whole (raw emulsion) *Control:* Avoidance *Regimen:* mixed *Duration:* 6 months *Maximum OIT dose:* 3.3 gm EWP (40 mL hens egg emulsion‐ 1 small egg)	**Desensitization** *Definition:* Neg DBPCFC *Maximum dose:* 40 mL HE emulsion 10–40 mL HE emulsion *Tested:* 6 months	**Desensitization** *ITT* 40 mL 6 months OIT 0/10 Cont 0/10 *ITT 10‐* 40 mL OIT 9/10 Cont 0/10	**Any** OIT 10/10 No data for avoidance **Adrenaline** OIT 0/10 C 0/10 **EoE** None found	Six months of SOTI with raw HE emulsion resulted in partial tolerance, with regular intake, in a significant percentage of children with severe egg allergy
**Escudero** 2015 Spain **SU**	Egg allergic patients consecutively recruited at the Department of Allergy, Hospital Infantil Universitario Nino Jesus in Madrid, Spain.	*Inclusion:* 1.Age 5–17 egg allergic on egg exclusion diet 2. history egg reactions 3. SPT (≥3 mm) and sIgE ≥0.7 kU/L for egg white (EW), ovalbumin (OVA) and/or ovomucoid (OVM), 4. Pos DBPCFC‐ dehydrated EW powder *Exclusion* 1.Severe anaphylaxis after egg ingestion 2.Egg non‐IgE mediated reactions 3.Immune deficiencies 4. CIs to adrenaline 5. Allergy to other components of challenge	*Total:* 61 *OIT:* 30 *Control:* 31 Age: 5–17yrs (med 8) (63% male) *OIT*: 30; 73% male *Cont*:31; 52% male	*Method:* Computerized generated randomization table in 1:1 ratio *Blinding:* None	*Intervention:* Hens egg white (Dehydrated) *Control:* Avoidance *Regimen:* mixed *Duration:* 3 months *Maximum OIT dose:* 2.8 gm EWP	**Desensitization** *Definition:* Neg DBPCFC (only for OIT group) *Cumulative dose:* 2.8 gm *Tested:* 3 months **Sustained Unresponsiveness** *Definition:* Neg DBPCFC *Cumulative dose:* 2.8 gm *Tested:* 4 months Time between OIT & OFC: 1 month	**Desensitization** *ITT* OIT 28/30 (93%) Control—postulated 1/31 (3%) **Sustained Unresponsiveness** *ITT* OIT 11/30 (37%) Control 1/31(3%)	**Any** OIT 21/30 C ? Adrenaline OIT 1/30 C not available **EoE** Not able to test in 2 patients‐ resolved	Demonstration of SU from a 3 month trial EW‐sIgE levels at the end of treatment predicted sustained unresponsiveness.
**Martín‐Muñoz** 2018 Spain SEICAP DS	The Spanish Society of Pediatric Allergy, Asthma and clinical Immunology (SEICAP) multicenter‐ 9 allergy units in the Spanish Public Health care system	*Inclusion:* 1. Age 6–9 years 2. Pos EW SPT>3 mm 3. EW sIgE >0.35KUA/L 4. Pos. DBPCFC *Exclusion:* 1. Severe or uncontrolled asthma 2. Severe atopic dermatitis 3. Esophagitis symptoms 4. Autoimmune, cardiovascular, or neuropsychiatric diseases 5. Beta blocker treatment 6. Food OIT past 12 months 7. Aeroallergen Immunotherapy in start‐up phase	*Total: 101* *OIT:* 76; PI 38 PII 38 *Control:* 25 Age—6–9 years	*Method:* centralized computer algorithm *Blinding:* None	*Intervention:* Hen's egg white (pasteurized) *Control:* avoidance *Regimen:* gradual 2 different: P1‐30% weekly and 5% daily updosing P2—30% weekly updosing *Duration:* 12 months *Maximum OIT dose:* 3.3 gm EWP randomized to daily or second daily ? NSAIDs as well	**Desensitization** *Definition:* Neg DBPCFC *Cumulative dose*:1 raw egg 3.3 gm EWP *Tested:* 12 months	**Desensitization** *ITT* OIT: 64/76 (84%) Control: 4/25 (16%)	**Any** OIT: 66/76 (86.8%) C: 8/25 (%) **Adrenaline** OIT 1/76 C 0/25 **EoE**‐ None found	PEW OIT is an effective treatment for children with persistent egg allergy. A 30% weekly plus 5% daily increment pattern could be more effective and safer than one with only 30% weekly increments.
**Itoh‐Nagato** 2018 Japan DS	9 allergy centres in urban areas in Japan	Inclusion: 1.5–15years 2. Hx IgE hen's egg allergy 3. sIgE ≥0.35Ua/ml 4. +ve DBPCFC ≤500 mg dried raw hen's egg white powder Exclusion: 1.Uncontrolled asthma 2. Uncontrolled atopic dermatitis 3.Grade 5 anaphylaxis on DBPCFC	Total:45 OIT: 23 Contol:22 Age: Median(range) OIT: 7(5–12) Control: 8(5–13)	Method Computer based 1:1 allocation Stratified by age, gender, sIgE, TD and grade of Sx at DBPCFC Blinding: None	Intervention: Whole egg lightly cooked Control: avoidance Regimen. Mixed Duration 3 months Maximum OIT: 1 gm EWP—1 whole scrambled egg	**Desensitization** Definition‐ neg DBPCFC Cumulative dose 1000 mg EWP Tested‐3 months	**Desensitization** ITT OIT 20/23 (87%) Control 5/22 (23%)	**Any** ITT 19/23%—83% C 0/22 **Adrenaline** OIT 2/23 (9%) C −0/22 **EoE**; 4 participants receiving OIT developed refractory GI tract symptoms‐ unable to investigate	
Cow's milk									
**Battista Panjo** 2010 Italy DS	Department of Pediatrics, Allergy Unit, Messina and Catania University hospitals	Inclusion: 1. Age: 4–10 years 2. Cows milk allergy from clinical history, SPT ≥3 mm, IgE specific antibodies and DBPCFC to cows milk Exclusion: 1.No allergy to soy (SPT, IgE or history) 2. Not sensitized to other foods	Total: 30 OIT: 15 Control: 15 Age: OIT 9 (4–12) Control: 10 (4–13)	Method: not supplied Blinding; DB	Intervention; whole cows milk Control: soy milk Regimen: Gradual Duration: 4 months Maximum; 200 mL whole milk (6.4 gm CM protein)	**Desensitization** Definition: Neg DBPCFC Cumulative dose: milk—200 mL Milk protein (4.6 gm)	**Desensitization** ITT OIT: 10/15 (75%) Control: 0/15 (0%)	**Any** OIT 10/15 C 0/15 **Adrenaline** OIT 2/15 C 0/15 **EoE**‐ not reported	3 had anaphylaxis at doses of 64 ml,4ml and 2 mL _ all these children were in the most severe group with symptoms elicited by 0.3–1 ml of milk at baseline
**Maeda** 2021 Japan ORIMA DS	Severe cows milk allergy (tol <=10 mL in OFC) Patients seen at: Dept of Pediatrics, Daisan Hospital, Jikei Univesity school of medicine Dept Pediatrics Showa University Hospital Aug 2011‐July 2016 with. Cows milk allergy	*Inclusion:* 1. Age: 3–12 years. 2. Cows milk allergy from clinical history, 3. IgE specific antibodies ≥0.7UA/ml 4. Lived within 30 min of hospital 5. Parents could be present for RUSH OIT 6. Consent given by child and/or parents and DBPCFC to cows milk 7. DBPCFC +ve (At least Sampson's Grade 2 symptoms after up to cumulative 10 mL. Cow's milk) (was 5 mL but changed after May 2013) *Exclusion:* 1.Hx of life threatening anaphylactic shock to cows milk 2. Uncontrolled bronchial asthma 3.Uncontrolled atopic dermatitis 4.Allergic to other foods (soybeans, chocolate, oats) 5. Physician judged ineligible because of complications 6. Difficulty withdrawing oral drugs for OFC	*Total:* 28 *OIT:* 14 *Control:* 14 *Age:* OIT: 5.5 +/− 2.4 yrs(SD) Control: 5.4 +/−2.3 yrs(SD)	*Method:* Randomization by independent data centre. Dynamic allocation with minimization adjusted for hospital, sex and cow's milk IgE level *Blinding* Not blinded	*Intervention:* Cow's milk *Control:* Elimination *Regimen:* mixed *Duration:* 1 year *Maximum:* 100 mL/day	**Desensitization** *Definition:* Neg DBPCFC *Cumulative dose:* 100 mL cow's milk	**Desensitization** ITT OIT: 7/14 (50%) Control:0/14 (0%)	**Any** OIT 12/14 C 3/14 **Adrenaline** OIT 7/14 (43%) Avoidance 0/14 **EoE**‐ not reported	
**Skripak** 2008 North Carolina USA DS	Paediatric allergy clinics at John Hopkin's University Hospital, Baltimore, Maryland, and Duke University Medical Centre, Durham, NC	*Inclusion:* 1. Children 6–21 years with known history of IgE‐mediated milk allergy 2. Pos SPT to milk extract (wheal ≥histamine control) or milk IgE levels >0.35kU/L 3. Pos milk challenge at baseline (cum 2.5 gm milk protein) *Exclusion:* 1. History anaphylaxis requiring hospitalization 2. History of asthma intubation 3. Current severe or persistent asthma	*Total:* 20 *OIT:* 13 *Control:* 7 *Age:* OIT median 9 yrs Control median 11 yrs	*Method:* randomized 2(OIT) to 1 (placebo)—no information on method *Blinding:* DB	*Intervention*: Powdered milk (non‐fat) *Control:* Placebo (maltodextran) *Regimen:* Gradual *Duration:* 3 months *Maximum:* 0.5 gm CM protein (15 mL milk)	**Desensitization** *Definition:* Neg DBPCFC *Cumulative dose:* milk protein 8 gm	**Desensitization** *ITT* OIT: 4/13 (13%) Control: 0/7 (0%)	**Any** OIT: 13/13 not available **Adrenaline** OIT 4/13 C 0/7 **EoE**‐ not reported	
**Dantzer** **2022** **USA** **DS**	John Hopkins Pediatric Allergy clinic, Baltimore	*Inclusion* 1. 3–18 yrs 2.Hx cow's milk reactivity 3. SPT≥3 mm neg control 4. sIgE >5kU/L 5. +ve DBPCFC ≤444 mg baked milk protein 6. Tolerate >3 mg baked milk protein *Exclusion* 1. Hx severe anaphylaxis 2. Severe/poorly controlled asthma 3. Poorly controlled AD *4.* Hx of eosinophilic oesophagitis past 3 yrs	*Total:* 28 *OIT:* 14 *Control*: 14 *Age:* both groups 9.5 median	Participants allocated 1:1 to Block randomization *Blinding:* DB	*Intervention* Baked milk powder *Control:* tapioca flour (in baked goods) *Regimen:* Mixed *Duration:* 52 weeks *Maximum:* 2000 mg baked milk protein	**Desensitization** *Definition:* ‐ve DBPCFC *Cumulative dose:* 4044 mg baked milk protein	**Desensitization** ITT *OIT* 11/15(73%) *Placebo* 0/15 (0%) Risk difference 73% Risk ratio 23.0 (95% CI 1.48–358) (0.5 added to each cell) NNT 1.5 (95% CI 1.1–2.2	OIT: AEs for 42% all doses (2222/5277) C: AEs 2% all doses. (94/5132) >95% of AEs in both OIT & placebo groups were rated “mild”. Any OIT 15/15 C 11/15 Adrenaline OIT 3/15 C 0/15 EoE: none reported	well tolerated although mild dosing‐related AEs were common in the OIT group. BMOIT induced a substantial level of desensitization after 12 months of treatment.

**TABLE 2:** Selection and loss of participants.

Study	Number excluded over assessed	Reason	Loss intervention	Reason	Loss control	Reason
Peanut						
Anagnostou (2014)	5/104	Did not meet inclusion criteria	6/49	1‐could not increase 1‐taste 2‐ frequent reactions 1‐ Persistent symptoms 1‐ no reason	4/50	3‐ did not want to be in control arm 1‐ Developed Crohn's disease
Bird	10/67	5‐withdrew consent 4‐tolerated 100 mg peanut protein on DBPCFC 1‐ serious anaphylaxis 1‐ reacted to placebo in DBPCFC	6/29	6‐ adverse events/compliance (mainly GI)	1/27	1‐ withdrew consent prior to treatment
PALISADE 2018	287/842	176 passed DBPCFC 39 withdrew consent 12 withdrawn by investigator 1‐ serious AE 59‐ another reason	102/416	51‐ adverse events 41 withdrew consent 1‐ investigator withdrawn 49 lost or other reason	11/139	3‐ adverse events 7‐ withdrew consent 1‐ another reason
Chinthrajah (2019)	120/152	7 passed DBPCFC 4 SPT <5 mm 21 declined	14/95	2‐ adverse events 4 non‐compliant 6 withdrew consent 1 investigator decision 1 lost	2/25	1 non‐compliant 1 withdrew consent
Blumchen (2018)	124/186	119 families refused 4 tolerant to peanuts 1 refused chocolate pudding	2/31	2 adverse events	7/31	2—adverse events 1‐ wrong study material 2 withdrawal of consent 1‐ non‐adherence 1 refused follow‐up DBPCFC
Hourihane (2020)	52/227	52‐ did not meet selection criteria	26/132	15‐ Adverse events 8‐ withdrew consent 2 lost to follow‐up 1‐ deviated from protocol	3/43	1‐ GI symptoms 1 withdrew consent 1 moved OS
Jones (2022)	63/209	63 did not meet eligibility criteria	28/96	2‐ couldn't updose 5 adverse events 18 withdrew consent 1 non‐compliance	27/50	4 adverse events (1 anaphylaxis) 14 withdrew consent 2 non‐compliance 1 lost 6 ?
Loke (2022)	47/248	14 passed DBPCFC 3 negative peanut SPT 16 withdrawn by investigator 6 consent withdrawn 5 reaction to placebo 1 taken probiotics 2 illnesses precluding participation	21/162	PPOIT 1 Adverse event 4 patient non compliant 2 parent decision 1 study inconvenience OIT 3 adverse events 3 patient non compliant 6 parent decision 1 other reasons	4/39	2 parent decision 1 relocated 1 study inconvenience 1 lost
Hen's egg						
Akashi (2017)	Information not provided		4/18	3 withdrew 1 refused second DBPCFC	2/18	2 refused second DBPCFC
Caminiti (2015)	29/59	Did not meet inclusion criteria	1/17	1 discontinued	3/14	1 lost 2 dropped out
Dello Iacono (2013)	6/26	1 parents unreliable 2 poorly controlled asthma 3 able to tolerate >0.9 mL raw egg on DBPCFC	10/10		10/10	
Escudero (2015)	30/91	21 declined to participate 9 did not meet inclusion criteria	5/30	2—adverse reactions 3 did not achieve desensitization in 3 months so did not perform challenge	0/31	
Martin‐Munoz (2017)	40/141	21 passed DBPCFC 8 refused DBPCFC ??“11 failed boiled and PEW” (not clear)	18/76	Not clear (5 refused food challenge)	3/25	Not clear
Itoh‐Nagato	3/48	Did not meet inclusion criteria 2 tolerated >500 mg dried EWP on DBPCFC A lot of anaphylaxis	3/23	1 failure to increase dose 2 Adverse events (A lot of anaphylaxis, 1 GIT)	1/22	1—adverse event on DBPCFC‐ withdrawn
Cow's milk						
Battista Panjo (2010)	19/49	19 did not meet inclusion criteria	5/15	2 stopped desensitization (unrelated to intervention) 3 stopped ‐severe Adverse events	1/15	1 stopped (not related to intervention)
Maeda (2021)	6/34	4 passed DBPCFC 1 poor physical condition 1 unable to ingest food at DBPCFC	2/14	2‐ adverse events/symptoms—unable to escalate dose	0/14	
Skripak (2008)	Information not provided		1/13	1‐ eczema flare, continuous	0/7	
Takahashi (2017)	2/18	2 passed DBPCFC	0/10		0/6	
Dantzner (2022)	11/41	11—passes DBPCFC	1/15	1 adverse effects	1/15	1‐ family member ill

#### 3.1.2 Intervention and intervention regimen

All trials used roasted peanut flour, three using AR101^29^, ^31^, ^32^ and one measuring and reporting specific Ara antigens (Table 3).^29^ All initial doses and dose increases were performed in clinical settings except for one study where the site and quantity of first day dosing and up‐dosing were not clear.^11^ *First day dose* The three trials using AR101^29^, ^31^, ^32^ gave escalating doses over the first day, building from 0.5 to 6 mg peanut protein. One trial escalated from 0.1 to 6 mg^27^ and one from 0.1 to 12 mg peanut protein.^26^ One study commenced with a dose of 2 mg^28^ and one titrated the initial dose to the food challenge eliciting dose (giving between 0.5 and 30 mg).^30^ *Up‐dosing regimen*. All studies increased doses fortnightly, and length of time to reach the maintenance dose varied between 4^26^ and 24^11^ months. *Final dose achieved* varied widely from 0.125^30^ to 4^11^ gm peanut protein. The AR101 studies^29^, ^31^, ^32^ had final doses of 0.3 gm peanut protein. *Maintenance*. Length varied from 0 to 18 months and total length of study treatment before assessment for DS varied from 6 to 24 months. *Sustained unresponsiveness*: one study^11^ measured SU at multiple time points (117, 130, 143 and 156 weeks). Total duration of intervention for this study was 24 months with a total of 12 months off OIT. A second study^26^ also measured SU 12 months after OIT completion (following 18 months of initial treatment). A third study^27^ measured SU 6 months after OIT completion (following 30 months of initial treatment).

**TABLE 3:** Oral Immunotherapy regimens.

Study	Exposure	Regimen type and dose	Resource use other meds	Final dose	Time to final or maintenance dose (months)	Tested at (months)
Peanut						
Anagnostou (2014)	Golden Peanut flour (defatted light roast)	Dosage‐ daily **First day**—2 mg peanut protein Gradual up‐dosing with 2 week increments to 800 mg/day (2, 5, 12.5, 25, 50, 100, 200, 400, 800 mg)	First dose and 2 weekly increases in clinical research facility	0.8 gm peanut protein	6	6
Bird (2018)	AR101 Peanut flour (defatted light roast).	Dosage‐ daily **First day**: escalation from 0.5 to 6 mg peanut protein Graduated doses in capsules12,20,40,80,120, 160,200,240, 300 mg	First day and Fortnightly up‐dosing in clinical setting	0.3 gm peanut protein	8	8.5
Blumchen (2018)	Lightly roasted peanut flour (Byrd Mill)	Dosage‐ daily First Day: Depended on eliciting dose (between 0.5 and 30 mg peanut protein 2 week up‐dosing	First day and Fortnightly up‐dosing in clinical setting	0.125–0.25 depending on eliciting dose	14	16
Chinthrajah (2019)	Peanut flour (Byrd Mill)	Dosage‐daily **First day** Gradual up‐dosing at home over 2 years 4000 gm/day then avoidance 4000 gm/day then 300 mg per day	Doses and setting not clear	4 gm peanut protein	24	24, 36
Hourihane	AR101	Dosage‐Daily **First day**—0.5–6 mg Second Day 3 mg 20–40 week up‐dosing from 3 to 300 mg—increased fortnightly	First (2 day) escalation phase initially under physician supervision at trial site Up‐dosing fortnightly at trial site	0.3 gm	5–9	9
Palisade	AR101	Dosage‐Daily First Day: 0.5–6 mg 2‐week gradual increasing from 3 to 300 mg	First Day supervised dose escalation	0.3 gm	6	12
Jones (2022)	Golden Peanut flour (defatted light roast)	Dosage—daily First day: 0.1–6 mg 2‐weekly dose increase to 2000 mg daily by week 30	First day and fortnightly dose increase in hospital.	2 gm	6	30, 36
Loke (2022) PPOIT‐003	1. 12% defatted peanut flour [50% peanut protein] 2. Probiotic (2 × 10^1^⁰ colony‐forming units of *L rhamnosus* ATCC 53103)	1. Dosage—daily First day: 0.1 mg to mg. 2‐weekly dose increase to 2000 mg. 2. Fixed dose daily probiotic or placebo given to all participants	First day and fortnightly increase in clinic	2 gm	4	18, 30
Hen's Egg						
Akashi (2017)	Whole egg powder (egg white protein conversion factor (EWPCF) 0.425)	Dosage ‐ daily **First Day:** 0.04 mg EWP Increasing ev 3–4 days: 0.09, 0.13, 0.27, 0.43, 0.64, 0.85, 1.23, 1.7, 2.55, 4.25, 6.34, 12.75, 21.25, 29.75, 42.50, 63.75, 85, 127.5, 212.5, 297.5, 425, 510, 637.5, 850, 1275, and 1700	OIT at home Antihistamines if recurrent symptoms	1.7 gm EWP (4 gm powder)	3	6
Caminiti (2015)	Dehydrated egg white (EWPCF = 1)	Dosage ‐ daily **First day** 0.1 mg EWP Weekly doubling: 0.2, 0.4, 0.8. 1.6, 3.2, 6.4, 12.8, 25.6, 51.2, 102.4, 204.8, 409.6, 819.2, 1638.4, 3276.8, 4000 mg	Weekly administration of doubled doses at hospital clinic	4 gm EWP	4	4
Della Iacono (2013)	Whole hen's egg raw emulsion (EWPCF 0.0825) 1 drop = 0.05 mls = 0.004 gm 40 mls = 3.3 gm)	Dosage‐ daily **First day**—4 mg Weekly increases: 8, 12, 17, 33, 37, 41, 50, 60, 100, 150, 170, 210, 410, 500, 600, 700, 740, 830, 1650, 1820, 1980, 2150, 2310, 2480, 3300 mg	First day and doubling doses at Weeks 1,4,9, 13, 19 in day hospital	3.3 gm EWP	6	6
Escudero (2015) SU	Dehydrated egg white (already provided in EWP)	Dosage—daily **First day** escalation from 0.08 to 140 mg EWP Weekly increments (depending on amount tolerated on first day) Then −188, 352, 1404 and 2808 mg of EW protein	Dose escalations on first and weekly increments in hospital	2.8 gm EWP	3	4
Martin‐Munoz (2019)	Pasteurized hens egg white 30 mls egg white has 4 gm EWP 1mls egg white = 0.133 gm = 133 mg	**First day**; initially 1 mL of 1/1000 solution of egg white, doubled until 0.4 mL undiluted egg white‐ 44 mg EWP Increasing in 2 different ways: P1‐30% weekly and 5% daily up‐dosing, then when target reached continue daily P2 –30% weekly up‐dosing—when target reached second daily	First day in hospital. Weekly increments performed in hospital	3.3 gm EWP	3.5	12
Itoh‐Nagato (2019)	Whole lightly cooked egg	**First day:** Initially 1/10 of threshold dose EWP, increased 1.2–1.5x at 309 min intervals, 3–5 times in 1 day. Allergic reactions determined subsequent dose When 1 gm EWP reached—equivalent to 8 gm raw egg white then scrambled egg heated to75‐80C for 10s Maintenance‐ daily ingestion for 2 months, then every other day next 4 months and more than once every 3 days thereafter	Rush phase to 1.0 gm EWP in hospital	1.0 gm EWP	3	3
Cow's milk						
Battista Panjo (2010)	Whole cow's milk	Dosage: daily First Day: 1 drop whole milk diluted 1:25 Doubling until week 18 Final dose‐ 200ml‐6.4 gm	First day and Weekly doubling in clinic till week 18	6.4 gm cow's milk protein (200 mL milk)	4	4
Skripiak (2008)	Non‐fat milk powder	Dosage: daily First day—0.4mg–50 mg Increasing 1–2 weekly to—500 mg(15 mL) whole milk	First day and weekly to two weekly up dosing in research setting	0.5 gm cow's milk protein	3	6
Maeda (2021)	Whole milk	Dosage: 3x day First 2 weeks—rush from 10^−4^ ml to 20 mL cows milk Then daily 19 weeks to 100ml‐ 3.2 gm	Hospitalized for rush 2 weeks	3.2 gm cow's milk protein (100 mL milk)	5	5
Dantzer (2022)	Baked milk protein	Dosage: daily First day‐ 0.1mg–25 mg (cumulative 44 mg) Increasing dose every 10–21 days 2000 mg.	First day and dose increases in clinic	2 gm baked milk protein	10	12

#### 3.1.3 Randomization & blinding

All studies used computer randomization with six using computer generated permuted blocks,^11^, ^26^, ^29^, ^30^, ^31^, ^32^ one^28^ used minimization based on age, sex, challenge threshold, sIgE, asthma severity and other food allergies, and one^30^ stratified by age and sIgE. In seven studies,^11^, ^26^, ^27^, ^29^, ^30^, ^31^, ^32^ participants were blinded to the intervention with control groups receiving placebo. In the remaining study, control group participants practiced peanut avoidance.^28^

#### 3.1.4 Meta‐analysis

**FIGURE 2:**
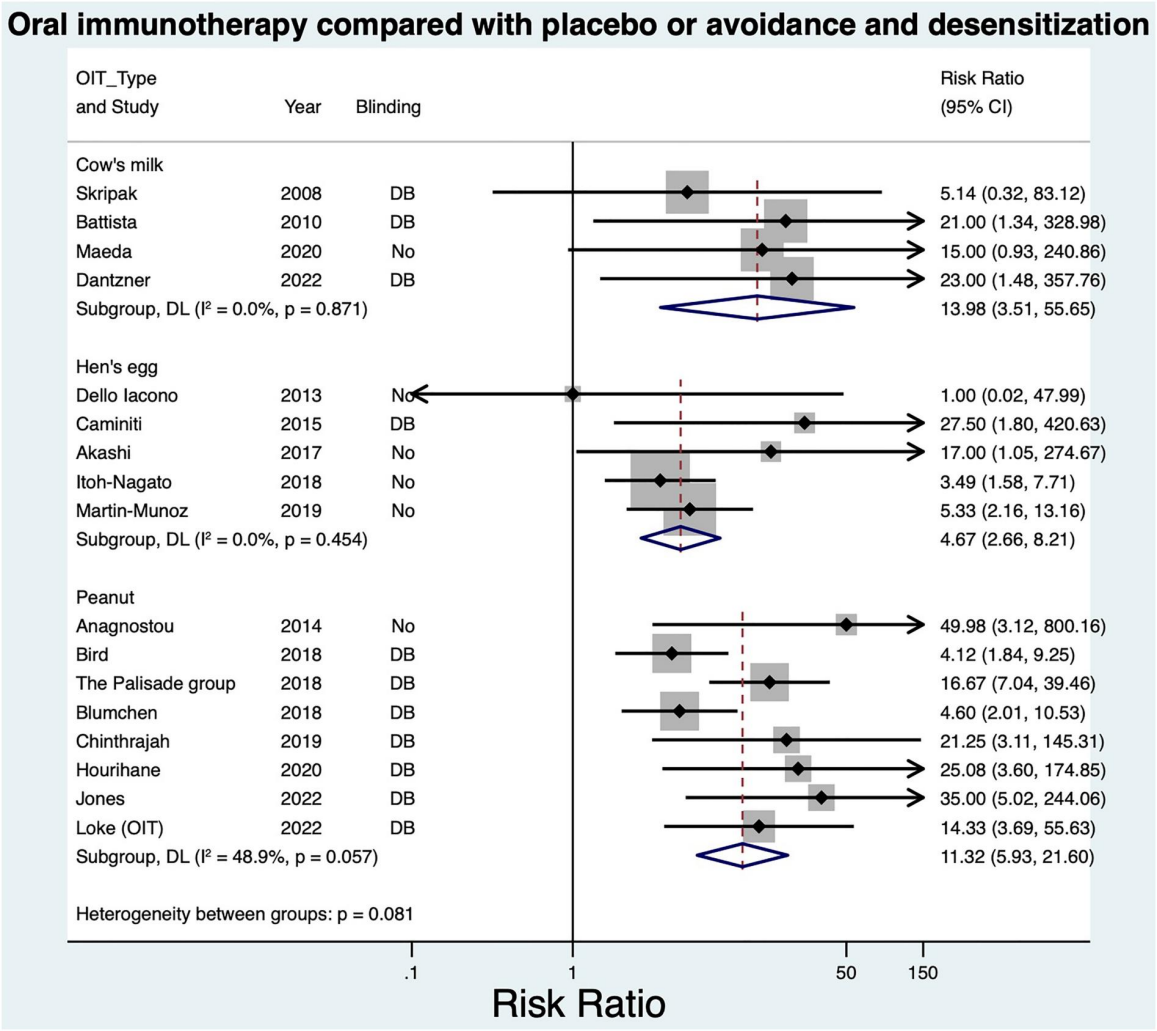
Meta‐analysis: Oral immunotherapy compared with placebo or avoidance and desensitization.

Meta‐analysis of eight OIT trials demonstrated increased DS in the intervention group: RR = 11.32; 95% CI 5.93–21.60, *I* ^2^ 48.9% (Figure 2).

**FIGURE 3:**
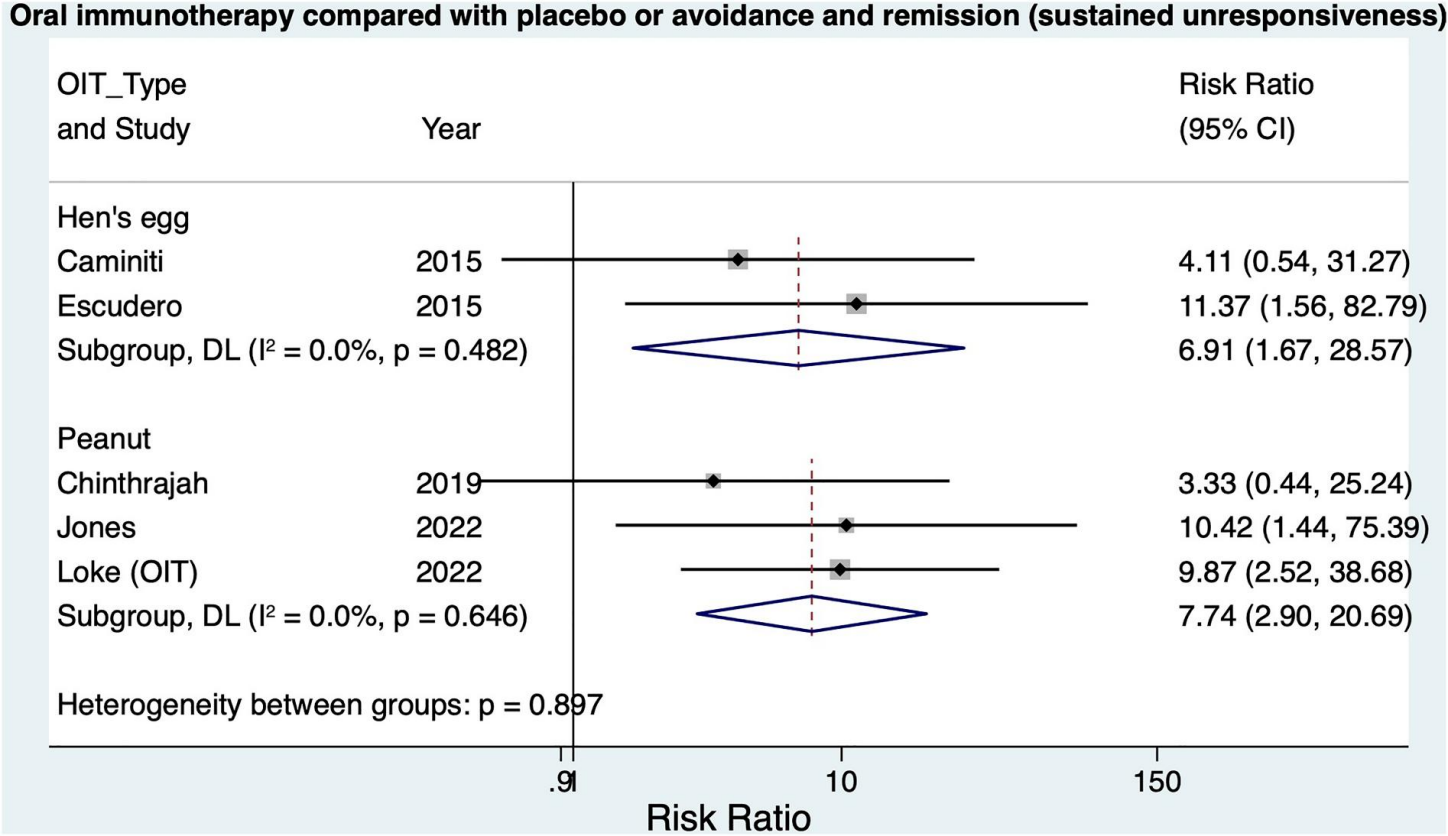
Meta‐analysis: Oral immunotherapy compared with placebo or avoidance and remission (sustained unresponsiveness (SU)).

Meta‐analysis of three OIT trials demonstrated increased SU in the intervention group: RR = 7.74; 95% CI 2.90–20.69, *I* ^2^ 0% (Figure 3).

From 8 studies, NNT(DS) = 1/(11.32–1) × 20/389 = 1.88. Therefore, based on the included studies and regimens, two people need to be treated for one to become desensitized.

From 3 studies; NNT (SU) = 1/(7.74–1) × 5/114 = 3.38 Therefore, based on the included studies and regimens, four people need to be treated for one to achieve SU after 6–12 months off intervention.

#### 3.1.5 Allergic adverse effects

**FIGURE 4:**
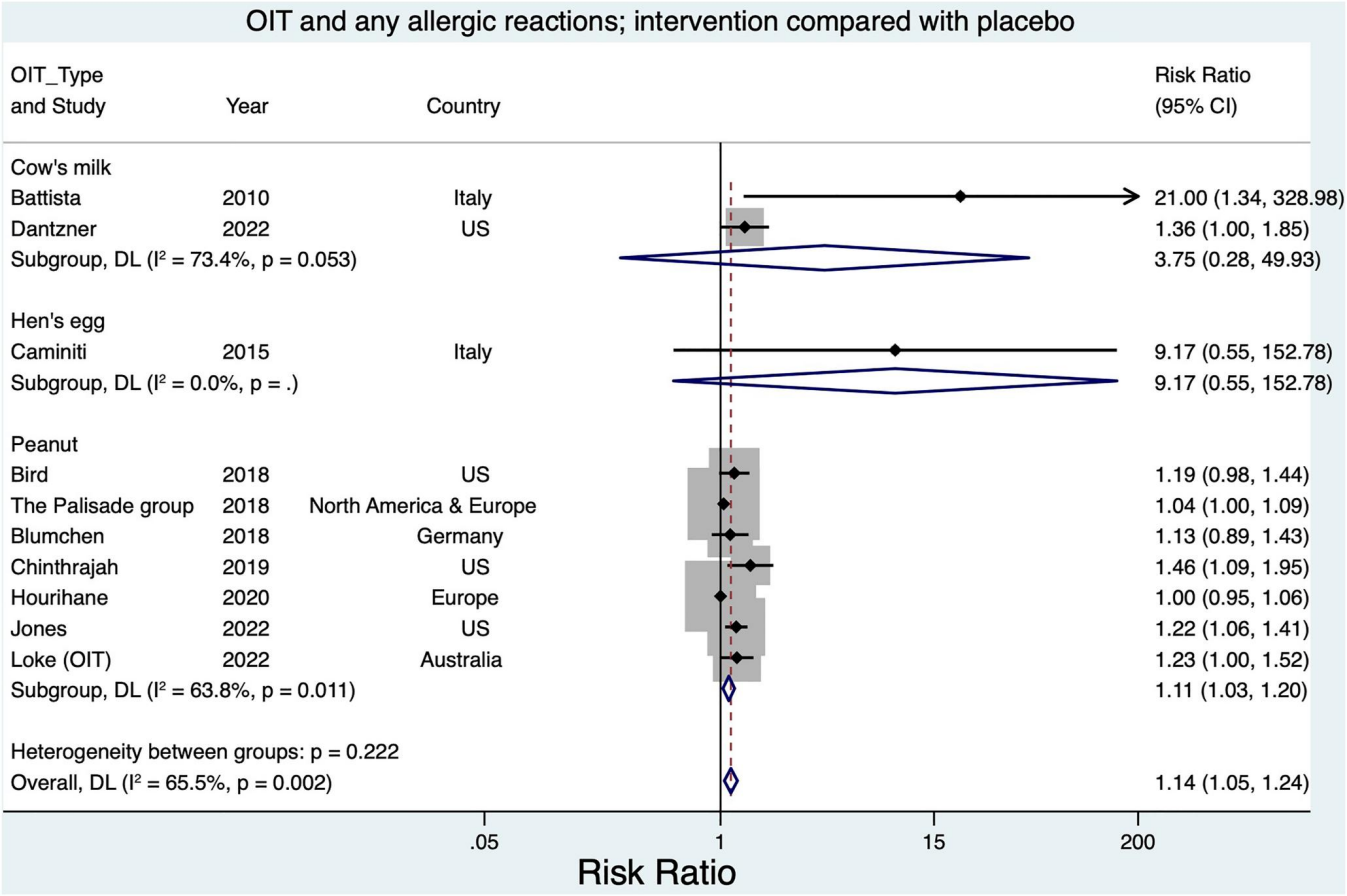
Oral immunotherapy and any allergic reactions.

**FIGURE 5:**
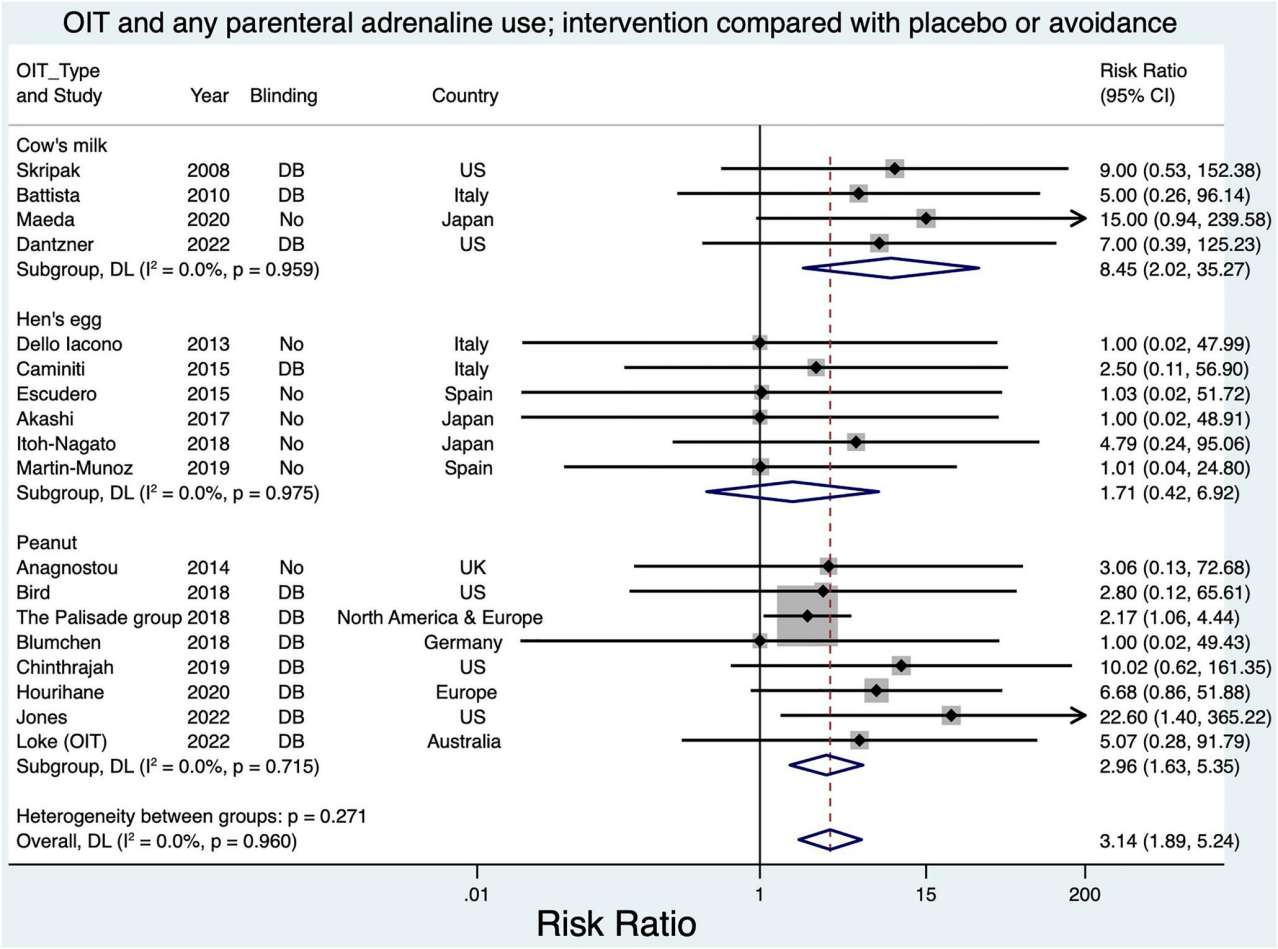
Oral immunotherapy and adrenaline use.

All participants had access to a health care facility, and health education was provided to the caretakers to manage allergic reactions (Figures 4 and 5). No children died or experienced permanent harm from allergic events related to the included trials. Allergic reactions were measured differently between trials. Some trials reported these as a rate per dose of immunotherapy, whilst others reported the number/proportion of children in intervention/control groups experiencing reactions; severity of these reactions was also classified differently.

All intervention trial arms experienced more adverse effects than control arms. In the non‐blinded studies with no placebo, food reactions (apart from severe reactions/anaphylaxis) in the control group were not recorded (as they were receiving no control intervention). Thus, we were not able to meta‐analyze adverse reactions for studies with no placebo group.

Meta‐analysis of any child having an allergic event from the seven double blind placebo‐controlled studies found a risk ratio of any allergic reaction for the intervention (compared to placebo) of 1.11; 95% CI 1.03, 1.20; however, heterogeneity was moderate (*I* ^2^ = 63.8%). Notably, the PALISADE trial^32^ (*N* = 555) identified no overall increase in allergic reactions associated with intervention (RR 1.09; 0.87, 1.36). However, in this trial,^32^ children in the active treatment group had an increased risk of parenteral adrenaline (RR 2.96; 95% CI 1.63, 5.35). Five studies^26^, ^27^, ^29^, ^30^ each reported 1–3 cases of eosinophilic esophagitis limited to treatment groups among those consenting to endoscopy.

NNT (adrenaline use) = 1/(2.96–1) × 9/366 = 20.75. Therefore, based on the included studies and regimens, 21 children need to be treated with peanut OIT for one to require at least one dose of adrenaline.

### 3.2 Hen's egg OIT

#### 3.2.1 Overview of studies

There were six RCTs of hen's egg OIT^24^, ^25^, ^33^, ^34^, ^35^, ^36^ from three countries: two from each country, that is, Japan^33^, ^35^; Italy^24^, ^34^; and Spain (Table 1).^25^, ^36^ All trials had less than 102 participants (range 20—101) with a mean of 49 and a total across all trials of 294. No trial included children below three or over 17 years of age. At baseline, three studies determined a reaction eliciting dose to investigate differences between treatment groups,^25^, ^33^, ^34^ and one study determined a reaction threshold to customize OIT.^35^ One study was restricted to a severely allergic group.^34^

Of the six RCTs, three excluded children with a history of severe anaphylaxis following egg ingestion or anaphylaxis at egg challenge^25^, ^33^, ^35^; three excluded children with poorly controlled or severe asthma^34^, ^35^, ^36^; two excluded children with severe eczema^35^, ^36^; two excluded children sensitized to foods other than egg^24^, ^34^; one excluded children with symptoms of esophagitis^36^; and two listed ability and/or willingness of families/participants to comply with the intervention as an inclusion criterion.^33^, ^34^, ^37^ From the three studies^34^, ^35^, ^36^ that provided a detailed breakdown, 12% (26/215) of the screened participants tolerated egg at the initial DBPCFC (Table 2).

##### Intervention and intervention regimen

Three trials used whole egg^33^, ^34^, ^35^ and three used egg white (Table 3).^24^, ^25^, ^36^ Three trials used dried/powdered egg preparations^24^, ^25^, ^33^ with others using emulsified raw egg^34^; pasteurized raw egg^36^ and lightly cooked egg.^35^ Intervention regimen differed between all studies.

*First day dose.* All studies except one^33^ administered the first dose in hospital or clinic. Three studies used in‐hospital dose escalation over 1 day^25^, ^35^, ^36^ with the first day final dose ranging between 44 mg^36^ and 1000 mg^35^ of egg white protein (EWP). Three studies started with low doses on the first day^24^, ^33^, ^34^ ranging from 0.01 mg^24^ to 4 mg^34^ EWP. *Up‐dosing regimen*. Three studies administered weekly^24^, ^25^, ^36^ increments in hospital/clinic. One study increased on a variable regimen and only performed these increases in hospital on weeks where the dose was doubled.^34^ One study administered gradual dose escalation at home^33^ and one study had no at home escalation phase.^35^ Length of time to achieve the final maintenance dose varied between a few days^35^ and 6 months.^34^ *Final dose achieved*. the final dose of EWP varied between 1.0 gm^35^ and 4 gm.^24^ *Maintenance*. Length varied from 0 to 8.5 months and total length of treatment before DS assessment varied from 4 to 12 months.

*Sustained unresponsiveness*: Two studies assessed SU. The first^24^ had an intervention duration of 4 months with SU assessed at 13 months (9 months off egg) and the second^25^ had an intervention duration of 3 months with SU assessed at 4 months (1 month off egg).

##### Randomization and blinding

All studies used computer algorithms to randomize participants. In one study,^24^ participants and investigators were blinded to the intervention with controls receiving corn flour.^24^ In the remainder, control groups practiced egg avoidance.

##### Meta‐analysis

Meta‐analysis of five egg OITs demonstrated increased risk of DS in the intervention group (Pooled risk ratio 4.67, (CI: 2.66–8.21, *I* ^2^ 0%) (Figure 2). Meta‐analysis of two trials demonstrated increased risk of SU in the intervention group; RR = 6.91 (1.67, 28.57), *I* ^2^ = 0% (Figure 3).

NNT (DS) = 1/(4.67–1) × 10/118 = 3.21 = 4. Therefore, based on the included studies and regimens, four people need to be treated for one to become desensitized.

NNT (SU) = 1/(6.91–1) × 2/45 = 3.9 = 4. Therefore, based on the included studies and regimens four people need to be treated for one to achieve SU.

##### Allergic adverse events

One placebo‐controlled egg trial found that the risk ratio for children experiencing any allergic events was 9.17 in the intervention compared to the control group (95% CI 0.55, 152.78) (Table 1 and Figure 4). In the other five studies with no placebo arm, 70 of 98 participants receiving intervention had some form of allergic reaction on at least one occasion.

From five of six studies where this information could be extracted, meta‐analysis of allergic events requiring adrenaline found a risk ratio for children experiencing allergic events (one or more) requiring parenteral adrenaline of 1.71 in the intervention group (95% CI 0.42, 6.92 *I* ^2^ 0%) (Figure 5).

NNT (adrenaline use) was not calculated as none of the children in the control groups were administered adrenaline.

### 3.3 Cow's milk OIT

#### 3.3.1 Overview of studies

Four trials^38^, ^39^, ^40^, ^41^ investigated cow's milk OIT: two from USA,^40^, ^41^ one from Japan,^38^ and one from Italy (Table 1).^39^ The number of participants varied from 20^40^ to 30^39^ with 106 in total. The average age of children ranged from 5.5^38^ to 9.5^39^, ^40^, ^41^ years. Three studies excluded children with severe or uncontrolled asthma,^38^, ^40^, ^41^ three excluded children with previous severe anaphylaxis,^38^, ^40^, ^41^ two excluded children with other food allergies^38^, ^39^ and two excluded children with severe atopic dermatitis.^38^, ^41^ The initial DBPCFC was passed by 18% (17/94) of those screened (where information was available) Table 2.

#### 3.3.2 Intervention regimen

Two trials used whole cow's milk^38^, ^39^ initially diluted with water, and one used non‐fat milk powder (Table 2).^40^ One study^41^ used baked cow's milk powder.

*First day dose:* One trial^39^ used a gradual introduction regimen with a first day dose of one drop of milk diluted by 1:25. Two studies^40^, ^41^ used rapid dose escalation on the first day—one^40^ from 0.4 to 50 mg cow's milk protein and the other^41^ from 0.1 to 25 mg. One study^38^ administered rush OIT in hospital for 2 weeks reaching a dose of 20 mL (0.64 mg cow's milk protein). *Up‐dosing regimen*. Three studies increased the dose weekly, fortnightly, or 3 weekly.^39^, ^40^, ^41^ The length of time to reach the maintenance dose varied between 3^40^ and 10^41^ months. *Final dose achieved*. One study^39^ had a maintenance dose of 200 mls of whole cow's milk (6.4 gm cow's milk protein); one^38^ had 100 mL (3.2 gm); one^41^ had 2 gm of baked milk protein; and one^40^ had 15 mL (0.5 gm cow's milk protein). *Maintenance*. Length varied from 0 to 3 months and total length of treatment before assessment for DS varied from 4 to 10 months. *Sustained unresponsiveness*: No studies.

#### 3.3.3 Randomization & blinding

In three studies^39^, ^40^ participants were blinded to the intervention with control groups receiving soy milk,^39^maltodextran^40^ or tapioca flour.^41^ Control participants in the remaining studies practiced avoidance.

#### 3.3.4 Meta‐analysis

Meta‐analysis of four OIT studies demonstrated increased desensitization in the intervention group: RR = 13.98 (95% CI 3.51–55.65) *I* ^2^ 0% (Figure 2).

NNT(DS) = not calculable.

None of the studies investigated remission.

#### 3.3.5 Allergic adverse effects

From two placebo‐controlled studies^39^, ^41^ where information was available, the pooled estimate of any allergic reaction for those taking the intervention was RR. 3.75; 95% CI 0.28, 49.93 *I* ^2^ 0% (Figure 3).

NNT(any allergic reaction) = 1/(3.75–1) × 14/29 = 0.75. Therefore, based on the included studies and regimens, on average one person needs to be treated for one to experience an allergic adverse effect.

From four trials, the risk of parenteral adrenaline use in the those receiving the intervention was RR 8.45; 95% CI 2.02, 35.27.*I* ^2^ 0% (Figure 4).

NNT (adrenaline use) = not calculable.

### 3.4 Risk of bias of included studies

Most studies were considered at low risk of bias with some being unclear concerning randomization procedures and one having excess loss to follow‐up (Table S4).^36^

## 4 DISCUSSION

Reviewing the most robustly designed RCTs, published in English, we found that OIT for peanut, egg, and milk had good efficacy for desensitization and some efficacy for remission. Allergic events were frequent in intervention groups with most being mild to moderate. NNT for adrenaline use was 1 in 20 people for peanut OIT.

Several methodological issues pertaining to RCTs of OIT may influence the quality of evidence. RCTs are considered the gold standard for determining whether interventions work because of their ability to deal with known and unknown confounders, leaving only causal associations. Despite this, certain criteria determine which RCTs are better able to address the question of OIT efficacy for inducing desensitization or remission. It is essential to establish that someone is food allergic using an OFC prior to starting therapy. In the included studies, despite careful participant selection, between 12% and 18% of all those screened were tolerant of the food being investigated. As food allergy can resolve without treatment, especially in younger children, a control group is essential and ideally this group should be treated with a placebo that mimics the intervention regimen. This is evidenced by 23% resolution of food allergy in Itoh‐Nagato et al's control group.^35^ Nevertheless, seven of 18 studies reviewed did not include a true placebo arm. Objective ascertainment of food allergy status prior to the trial is therefore essential, otherwise effects may be overestimated and the possibility that the child was not truly allergic at baseline cannot be excluded. The gold standard for objective measurement is the performance of a standardized double‐blind placebo‐controlled oral food challenge. Similarly, all participants must have objective ascertainment of food allergy status at the end of the trial using OFC. The most reliable RCT evidence comes from intention to treat analyses. Using per protocol analyses, especially in food allergy treatment, is likely to overestimate the efficacy of the intervention as participants who drop out of intervention arms are more likely to be food allergic than those who remain.

We found only eight peanut, six hen's egg, and four cow's milk RCTs that met our inclusion criteria of objective ascertainment of food allergy by OFC at the beginning and end of the trial for both intervention and control groups and analysis by intention to treat.

We found that, on average, two people needed to be treated with OIT for one to be desensitized to peanut (from eight studies), and four needed to be treated for one to be desensitized to egg (from five studies). For remission, with limited studies, NNTs were four for egg (two studies) and 4 for peanut (3 studies).

Three recent systematic reviews of OIT were published in 2022. They have all captured different studies in their inclusion criteria, so they have differing numbers of studies and different estimates for efficacy and harm. For example, for milk OIT efficacy meta‐analyses, one review^22^ found a RR of 7.35 (2.82–19.13) from 11 studies, a second review^20^ found a RR of 12.3, 95% CI: 5.9 to 26.0 from 13 studies, and the third^21^ RR 5.7 (1.9–16.7) from 8 studies. These differing estimates are confusing for patients, clinicians and for informing guidelines. As can be seen from this example, criteria for study inclusion are critical for obtaining the most accurate estimates of efficacy and harm, to inform guidelines and accessibility of this promising but expensive and potentially harmful therapy.

Efficacy must be balanced with the rate of allergic events. Although allergic reactions were generally mild and easily managed, most children receiving the intervention had an allergic reaction, and many had repeated reactions. To bring the evidence from all included studies together, we reported the proportion of subjects experiencing reactions. However, this is a crude measure of safety. Exposure adjusted incidence of adverse events (AE) is more meaningful as it describes the frequency or burden of AEs and offers a more precise measure of safety. In this review, we found OIT was associated with parenteral adrenaline use for every 20 patients treated with peanut OIT.

It is concerning that in four of the eight included peanut OIT trials there were between one and three diagnosed cases of eosinophilic oesophagitis (EoE) (total 6 across 4 trials). A 2014 meta‐analysis^42^ identified that EoE may occur in up to 2.7% of those undergoing OIT. A more recent, systematic review^13^ on 12 trials comprising 640 participants published between Jan 2019 and Jan 2020 found that 3.9% of participants withdrew from OIT because of gastro‐intestinal adverse effects, and two of these (0.3% of the 640 participants) were diagnosed with EoE.

After inadvertent or overt ingestion of similar quantities of allergenic food, some individuals develop anaphylaxis, whilst others experience milder symptoms such as mouth tingling or abdominal cramps.^43^ There were differences in study populations for the allergic threshold of included participants. Most studies excluded participants with related disorders such as asthma or atopic dermatitis or those with multiple food allergies. Only two of the included studies^34^, ^44^ investigated OIT in multi‐allergic children, limiting generalizability to children with specific risk profiles. These studies suggest that the ability to achieve DS and remission in highly allergic children may differ from less allergic children and regimens may need modification in dosage and length of intervention or the addition of an immunomodulatory agent. Given differing reactivity thresholds, low dose and/or high dose OIT may only be suitable for specific subgroups of people with food allergy.

The number of children maintaining long‐term remission is not clear. The recent peanut trial by Chiinthrajah et al.^11^ investigated long‐term remission in participants achieving DS at 2 years, finding remission waned over time, from 35% at 3 months to 13% at 1 year after the intervention had stopped.

Whether QoL is improved by OIT is an important part of the decision for undertaking OIT. Three of our included studies^26^, ^31^, ^35^ investigated changes in QoL. One placebo‐controlled study^31^ of peanut OIT found improvements in all domains of self‐reported and food allergy quality of life. However, unblinding was performed just prior to the second QoL assessment, which may have influenced responses. A more recent placebo‐controlled study found good evidence in the improvement of QoL in the treatment group.^26^ The third study^35^ which compared intervention to avoidance, found increased QoL in those receiving OIT. Other QoL research has produced mixed findings.^16^ ^,^ ^17^

The goals of OIT may differ depending on the individual child, their number of food allergies and their degree of reactivity or threshold dose for reaction. Although patients and physicians want a long‐term solution such as remission of food allergy, families of food allergic children may have more modest goals including the desire to reduce the likelihood of anaphylaxis in the case of inadvertent ingestion of trace amounts of allergen, or to tolerate small amounts of the allergen that might be encountered through trace contamination of food products.^45^

The strength of our approach is that we performed a high‐quality objective process to choose the best RCTs to provide the most accurate evidence. However, this limited our studies to small, highly select populations. Populations included in RCTs are often not representative of the intended target population. Typically, most participants included in the trials did not have other food allergies, had not had a previous severe anaphylaxis, and did not have severe or uncontrolled asthma. Additionally, entry to many trials required highly supportive parents. The population of food allergic individuals included in these RCTs may be very different from the population in which the proposed treatment is to be applied. Therefore, the efficacy found in these trials may not be the same as the possible effectiveness that could be achieved in a real‐world population of food allergic children. Furthermore, the rate and outcomes of adverse allergic events experienced during OIT may differ in families that are not as highly motivated to complete OIT and follow safety guidelines. In addition, study regimens were highly heterogenous and the overall number of participants was relatively low. Despite this, there was little statistical heterogeneity when studies were pooled in meta‐analyses.

More research is needed with standard interventions and regimens to provide greater certainty around the efficacy and safety of OIT and to understand how to achieve and maintain greater levels of remission.^46^ Further research should address the risks of eosinophilic esophagitis and whether the risk is greater for specific children, and whether OIT risks can be mitigated by concurrent immunomodulatory agents or probiotics.^26^, ^47^ We also need further understanding of the impact of OIT on QoL for food allergic children and their families.

## 5 CONCLUSION

This systematic review of high‐quality RCTs found that OIT had good efficacy in inducing desensitization and some efficacy for remission despite considerable heterogeneity in study methodology. We also confirmed that allergic events occurred frequently during OIT. Participants included in these RCTs are not representative of the entire food allergy population and care must be taken applying these findings to other populations where both efficacy and adverse effects may differ. Decisions around undertaking OIT need to be informed by consideration of individual circumstances.

## AUTHOR CONTRIBUTIONS

All authors were involved in the initial conception of this review and protocol development. Nilakshi Waidyatillake, Merryn Netting, Rachel L. Peters, Xin Dai and Caroline J. Lodge contributed to article selection, data extraction, and risk of bias assessments. Writing of the manuscript was led by Caroline J. Lodge and Nilakshi Waidyatillake with input from all authors. All authors have contributed to and sighted the final version.

## CONFLICT OF INTEREST STATEMENT

Caroline J. Lodge and Shyamali C. Dharmage have received investigator‐initiated grants from GSK and Astra Zeneca for unrelated work and are both supported by NHMRC investigator grants; Kirsten P. Perrett is supported by a NHMRC fellowship/Melbourne Children's Clinician‐Scientist Fellowship and has received unrelated funding from Aravax, DBV Technologies, Novartis and Siolta and consultant fees from Aravax; Mimi L. K. Tang has received speaker fees from Nestle Health Science, is a consultant to Bayer Pharmaceuticals and has received research funding from NHMRC, Bayer Pharmaceuticals, Abbot Nutrition, and Prota Therapeutics; Merryn Netting, John Burgess, Jennifer J. Koplin, Rachel L. Peters, Catherine J. Hornung, Nilakshi Waidyatillake, Xin Dai have no conflicts of interest to declare.

## Supporting information

Supporting Information S1
