# Supplementary material for: Efficacy and safety of oral immunotherapy for peanut, cow's milk, and hen's egg allergy: A systematic review of randomized controlled trials
Source: Clin Transl Allergy. 2023 Jul 1;13(7):e12268. doi: 10.1002/clt2.12268 (PMC10314278; doi:10.1002/clt2.12268)
Supplement: Supplementary file 1 — Supporting Information S1 [file CLT2-13-e12268-s001.docx]

Supplementary material

Title: Efficacy and Safety of oral immunotherapy for peanut, cow’s milk and hen’s egg allergy: a systematic review of randomized controlled trials

Short title: Efficacy/safety of oral immunotherapy for food allergy

A/Prof Caroline J Lodge^1,2^ PhD, Dr Nilakshi Waidyatillake^1^ PhD, A/Prof Rachel L Peters ^2,3^ PhD, Dr Merryn Netting^2,4,5^ PhD, Dr Xin Dai^1^PhD, Dr John Burgess^1,2^PhD, Dr Catherine J Hornung^2^ PhD, A/Prof Kirsten Perrett^2,3,4^PhD, Prof Mimi LK Tang^2,3,4^,PhD *A/Prof Jennifer J Koplin^2,3^PhD, *Prof Shyamali C Dharmage^1,2^PhD

- Equally contributed senior

*^1^Allergy and Lung Health Unit, Melbourne School of Population and Global Health, The University of Melbourne, Carlton, VIC, 3053, Australia.*

*^2^Centre for Food and Allergy Research, Murdoch Children’s Research Institute, Parkville, VIC, 3052, Australia.*

^3^*Department of Pediatrics, The University of Melbourne, VIC, 3052, Australia.*

^4^*Department of Allergy and Immunology, Royal Children’s Hospital, Melbourne, VIC, 3052, Australia*.

^5^*Women and Kids Theme, South Australian Health and Medical Research Institute (SAHMRI), Adelaide, South Australia, Australia.*

^6^*Discipline of Paediatrics, University of Adelaide, Adelaide, South Australia, Australia*

Correspondence to: A/Prof Caroline Lodge, ^1^Allergy and Lung Health Unit, Melbourne School of Population and Global Health, The University of Melbourne, Carlton, VIC, 3053, Australia email: clodge@unimelb.edu.au

Table S1 EMBASE Search terms

| **EMBASE Advanced search** | |
| --- | --- |
|  |  |
| 1 | randomized controlled trial/ |
| 2 | randomized controlled trial.mp. [mp=title, abstract, heading word, drug trade name, original title, device manufacturer, drug manufacturer, device trade name, keyword, floating subheading word, candidate term word] |
| 3 | "randomized controlled trial".mp. [mp=title, abstract, heading word, drug trade name, original title, device manufacturer, drug manufacturer, device trade name, keyword, floating subheading word, candidate term word] |
| 4 | RCT.mp. [mp=title, abstract, heading word, drug trade name, original title, device manufacturer, drug manufacturer, device trade name, keyword, floating subheading word, candidate term word] |
| 5 | randomised controlled trial.mp. [mp=title, abstract, heading word, drug trade name, original title, device manufacturer, drug manufacturer, device trade name, keyword, floating subheading word, candidate term word] |
| 6 | 1 or 2 or 3 or 4 or 5 |
| 7 | oral immunotherapy/ |
| 8 | oral immunotherapy/ or immunotherapy.mp. |
| 9 | immunotherapy.mp. |
| 10 | oral immunotherapy/ or active immunotherapy/ |
| 11 | OIT.mp. [mp=title, abstract, heading word, drug trade name, original title, device manufacturer, drug manufacturer, device trade name, keyword, floating subheading word, candidate term word] |
| 12 | 7 or 11 |
| 13 | 6 and 12 |
| 14 | food allergy/ |
| 15 | food allergy.mp. |
| 16 | food hypersensitivity.mp. |
| 17 | *sensitization/ |
| 18 | sensitization.mp.­­­­ |
| 19 | 14 or 15 or 16 or 17 or 18 |
| 20 | 13 and 19 |
|  |  |

Table S2 Pubmed Search terms

| **PubMed advanced search** | |
| --- | --- |
| Sarch | Query |
| #38 | Search ((((food allergy[MeSH Terms]) OR "food allergy") OR food hypersensitivity[MeSH Terms])) AND ((((((immunotherapy[MeSH Terms]) OR "oral immunotherapy")) AND ((((randomized controlled trial[MeSH Terms]) OR RCT) OR "randomized controlled trial") OR "randomised controlled trial"))) AND (((((food) OR nut) OR peanut) OR milk) OR egg)) Sort by: PubDate |
| #37 | Search ((food allergy[MeSH Terms]) OR "food allergy") OR food hypersensitivity[MeSH Terms] Sort by: PubDate |
| #36 | Search food allergy[MeSH Terms] Sort by: PubDate |
| #35 | Search "food allergy" Sort by: PubDate |
| #34 | Search food hypersensitivity[MeSH Terms] Sort by: PubDate |
| #29 | Search hypersensitivity[MeSH Terms] Sort by: PubDate |
| #28 | Search (((((immunotherapy[MeSH Terms]) OR "oral immunotherapy")) AND ((((randomized controlled trial[MeSH Terms]) OR RCT) OR "randomized controlled trial") OR "randomised controlled trial"))) AND (((((food) OR nut) OR peanut) OR milk) OR egg) Sort by: PubDate |
| #27 | Search ((((food) OR nut) OR peanut) OR milk) OR egg Sort by: PubDate |
| #26 | Search food Sort by: PubDate |
| #25 | Search nut Sort by: PubDate |
| #24 | Search peanut Sort by: PubDate |
| #23 | Search milk Sort by: PubDate |
| #22 | Search egg Sort by: PubDate |
| #21 | Search (((immunotherapy[MeSH Terms]) OR "oral immunotherapy")) AND ((((randomized controlled trial[MeSH Terms]) OR RCT) OR "randomized controlled trial") OR "randomised controlled trial") Sort by: PubDate |
| #20 | Search (immunotherapy[MeSH Terms]) OR "oral immunotherapy" Sort by: PubDate |
| #19 | Search immunotherapy[MeSH Terms] Sort by: PubDate |
| #17 | Search oral immunotherapy[MeSH Terms] Sort by: PubDate |
| #16 | Search "oral immunotherapy" Sort by: PubDate |
| #15 | Search oral immunotherapy Sort by: PubDate |
| #14 | Search OIT Sort by: PubDate |
| #13 | Search (((randomized controlled trial[MeSH Terms]) OR RCT) OR "randomized controlled trial") OR "randomised controlled trial" Sort by: PubDate |
| #12 | Search randomized controlled trial[MeSH Terms] Sort by: PubDate |
| #8 | Search (randomised controlled trial[MeSH Terms]) Sort by: PubDate |
| #5 | Search randomised controlled trial[MeSH Terms] Sort by: PubDate |
| #7 | Search asthma[MeSH Terms] Sort by: PubDate |
| #6 | Search RCT[MeSH Terms] Sort by: PubDate |
| #4 | Search RCT Sort by: PubDate |
| #3 | Search "randomized controlled trial" Sort by: PubDate |
| #2 | Search "randomised controlled trial" Sort by: PubDate |
| #1 | Search randomised controlled trial Sort by: PubDate |

Table S3 Cochrane Search terms

ID Search Hits

#1 oral immunotherapy in Trials

#2 OIT in Trials

#3 #1 or #2 in Trials

#4 food hypersensitivity

#5 food allergy

#6 food sensitization

#7 #4 or #5 or #6

#8 #7 and #3

Table S4 Risk of Bias

| Author, year | Randomization Process | Deviations from the intended intervention | Missing outcome data | Measurement of the outcome | Selection of the reported result | Overall risk of bias | Support judgement |
| --- | --- | --- | --- | --- | --- | --- | --- |
| Akashi et al. 2017 | Some concerns | Low | Low | Low | Low | Some concerns | -Cumulative dose egg tolerated at baseline higher in intervention group ( 600mg vs 200 mg) |
| Anagnostou et al. 2014 | Low | Low | Low | Low | Low | Low | - |
| Battista- panjo et al. 2010 | Some concerns | Low | Low | Low | Low | Some concerns | There is no information for randomized sequence |
| Bird et al. 2017 | Low | Low | Low | Low | Low | Low | - |
| Blumchen et al. 2018 | Low | Low | Low | Low | Low | Low | - |
| Caminiti et al. 2015 | Some concerns | Low | Low | Low | Low | Some concerns | There is no information for randomized sequence |
| Chinthrajah et al 2019 | Low | Low | Low | Low | Low | Low | - |
| Dantzner et al 2022 | Low | Low | Low | Low | Low | Low |  |
| Della Iacono et al. 2013 | Low | Low | Low | Low | Low | Low | - |
| Escudero et al. 2015 | Low | Low | Low | Low | Low | Low | - |
| Itoh-Nagato et al 2018 | Low | Low | Low | Low | Low | Low | - |
| Hourihane et al 2020 | Low | Low | Low | Low | Low | Low | - |
| Jones et al 2022 | Low | Low | Low | Low | Low | Low |  |
| Loke et al  2022 | Low | Low | Low | Low | Low | Low |  |
| Maeda et al. 2020 | Low | Low | Low | Low | Low | Low | - |
| Martin-munoz et al. 2019 | Low | High | High | Low | Low | High | -Study has high proportion of participants lost to follow-up or refused to continue. |
| Palisade group 2018 | Low | Low | Low | Low | Low | Low | - |
| Skripak et al. 2008 | Some concerns | Low | Low | Low | Low | Some concerns | There is no information for randomized sequence. |
| Takahashi et al 2017 | Some concerns | Low | Low | Low | Low | Some concerns | There is no information for randomized sequence. |
